# High-Performance Few-layer Mo-doped ReSe_2_ Nanosheet Photodetectors

**DOI:** 10.1038/srep05442

**Published:** 2014-06-25

**Authors:** Shengxue Yang, Sefaattin Tongay, Qu Yue, Yongtao Li, Bo Li, Fangyuan Lu

**Affiliations:** 1 State Key Laboratory of Superlattices and Microstructures, Institute of Semiconductors, Chinese Academy of SciencesP.O. Box 912, Beijing 100083, China; 2 School for Engineering of Matter, Transport and Energy, Arizona State University, Tempe, AZ 85287, United States; 3 College of Science, National University of Defense Technology, Changsha 410073, China

## Abstract

Transition metal dichalcogenides (TMDCs) have recently been the focus of extensive research activity owing to their fascinating physical properties. As a new member of TMDCs, Mo doped ReSe_2_ (Mo:ReSe_2_) is an octahedral structure semiconductor being optically biaxial and highly anisotropic, different from most of hexagonal layered TMDCs with optically uniaxial and relatively high crystal symmetry. We investigated the effects of physisorption of gas molecule on the few-layer Mo:ReSe_2_ nanosheet based photodetectors. We compared the photoresponse of the as-exfoliated device with annealed device both in air or ammonia (NH_3_) environment. After annealing at sub-decomposition temperatures, the Mo:ReSe_2_ photodetectors show a better photoresponsivity (~55.5 A/W) and higher EQE (10893%) in NH_3_ than in air. By theoretical investigation, we conclude that the physisorption of NH_3_ molecule on Mo:ReSe_2_ monolayer can cause the charge transfer between NH_3_ molecule and Mo:ReSe_2_ monolayer, increasing the n-type carrier density of Mo:ReSe_2_ monolayer. The prompt photoswitching, high photoresponsivity and different sensitivity to surrounding environment from the few-layer anisotropic Mo:ReSe_2_ can be used to design multifunctional optoelectronic and sensing devices.

Two-dimensional (2D) materials are an emerging class of new materials with exotic properties and great promise for use in next-generation nanoelectronic devices. The most studied 2D materials, graphene, has shown exceptional physical, chemical, optical, magnetic and mechanical properties^1^,^2^,^3^,^4^,^5^,^6^,^7^. Graphene-based optoelectronic devices not only operate in a very wide wavelength range^8^,^9^, but also show extremely fast carrier transport (approaching *ca.* 200000 cm^2^ V^-1^ s^-1^ for a free sheet)^2^,^10^. Despite graphene's superior properties, graphene is a zero-gap semimetal, and the lack of optical band gap limits its applications^11^. Therefore, the research based on other 2D materials with an intrinsic band-gap has been triggered.

Photodetector is an optoelectronic device that absorbs light with a certain wavelength and generates electron-hole pairs, and then produces electrical signal due to separation and directional movement of the electron-hole pairs. In recent years, photodetectors have made remarkable progress driven by urgent needs in numerous applications, such as flame detection, engine monitoring, missile plume detection, chemical/biological sensing, and intersatellite communications^12^,^13^,^14^,^15^,^16^,^17^. It is now widely recognized that nanostructured semiconductors, in comparison to bulk materials, may provide better photodetection performance due to their large surface area, low dimensions and size dependent properties, such as increased photon absorption, enhanced charge separation and migration, and surface sensitivities^18^,^19^. Some one-dimensional (1D) semiconducting nanostructures have been utilized to design photodetectors, but preparation of these 1D nanomaterials is usually complex^20^. 2D materials are relatively easy to fabricate circuits and some complex structures. The very high surface-to-volume ratio of single- or few-layer 2D materials enables promoted charge separation and highly light sensitivity. Graphene recently has been used for fabricating photodetectors^21^. However, graphene-based photodetectors are limited by their low responsivity (~10^−2^ AW^−1^), low external quantum efficiency (EQE) (0.1–0.2%), and lack of spectral selectivity^20^,^22^. Therefore, other 2D materials have been explored for enhancing responsivity and spectral selectivity of photodetectors. Very recently, semiconducting TMDCs with the common formula MX_2_, where M indicates a transition metal (M = Mo, W, V, Nb, Ta, Ti, Zr, Hf, Re) and X represents a chalcogen (Se, S or Te), emerged with great research interests^23^. GaS nanosheet photodetectors made on SiO_2_/Si substrates or flexible polyethylene terephthalate (PET) substrates exhibit a photoresponsivity at 254 nm of up to 4.2 AW^−1^ and 19.2 AW^−1^, respectively, which far exceeds that of graphene-based devices. The reduction of the effective mass at the valence band maximum with decreasing layer thickness enhances the carrier mobility of the GaS nanosheets, contributing to the high photocurrent^20^. An ultrasensitive monolayer MoS_2_ phototransistor shows a maximum external photoresponsivity of 880 AW^−1^ at the wavelength of 561 nm. This is due to their improved mobility, as well as the contact quality and positioning technique^23^.

Most of the 2D layered materials, such as graphene, MoS_2_, WS_2_ and WSe_2_ et al. have highly crystal symmetry. Therefore, the photonic, electronic, and mechanical properties of these 2D materials are largely isotropic and almost do not depend on the change of direction. In fact, the anisotropic properties of 2D materials have rarely been explored before for novel optoelectronic and electronic device applications. Mo:ReSe_2_, a new member of TMDCs, is an anisotropic semiconductor crystallized in a distorted layered CdCl_2_-type octahedral structure of triclinic symmetry, different from most of hexagonal layered TMDCs. A clustering of Re_4_ diamond units forms along the *b*-axis within the van der Waals plane in Mo:ReSe_2_ monolayer, resulting in the crystals being optically biaxial^24^. However, other TMDCs with hexagonal structure (e.g. 2H–MoS_2_ and MoSe_2_) are optically uniaxial with their optical axis perpendicular to the van der Waals plane^25^. On account of “diamond chains” clustering structure, Mo:ReSe_2_ shows in-plane optical and electrical anisotropic response, which may be exploited for fabrication of polarization sensitive photodetectors, photoelectrochemical solar cells and other optoelectronic devices^26^,^27^.

In this communication, we report the few-layer Mo:ReSe_2_ nanosheets based photodetectors. These few-layer Mo:ReSe_2_ nanosheets were mechanically exfoliated on SiO_2_/Si substrates, and were characterized by atomic force microscopy (AFM) and Raman spectra. Two-terminal photodetectors were fabricated with deposition of Au electrodes. We compare the photoresponse of the as-exfoliated devices with annealed devices both in air or NH_3_ environment. After annealing at sub-decomposition temperatures, the Mo:ReSe_2_ photodetectors show a better photoresponsivity and higher EQE. Especially, the devices operated in NH_3_ show higher performance than in air. The response time of device is also less than 100 ms. The prompt photoswitching, high photoresponsivity and different sensitivity to surrounding environment from the anisotropic Mo:ReSe_2_ nanomaterials pave an avenue to multifunctional optoelectronic and sensing device applications with 2D semiconductors.

## Results

**Figure 1:**
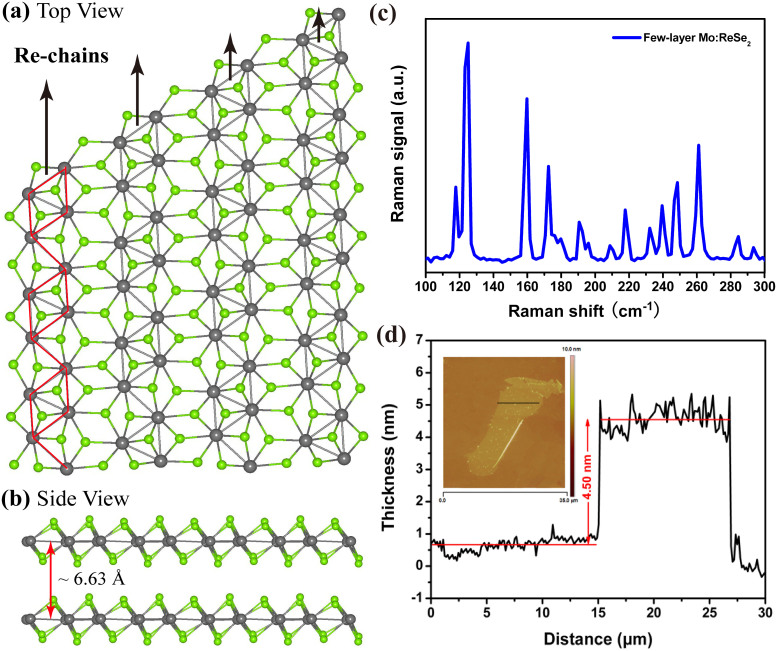
(a) The top view and (b) side view of Mo:ReSe_2_ nanosheet, (c) Raman spectrum and (d) AFM image of the few-layer Mo:ReSe_2_ nanosheet.

Figure 1a shows the structure of a single layer of Mo:ReSe_2_ nanosheet. The 1T-Mo:ReSe_2_ consists of edge-shared MX_6_ octahedra. The movement of the Re atoms toward each other forms a Re_4_ diamond unit which is coplanar and coupled with one another to comprise a clustering pattern of diamond chains, resulting in a lattice distortion^24^. The layer thickness of Mo:ReSe_2_ is ~6.6 Å (Figure 1b). Due to the reduced crystal symmetry, Mo:ReSe_2_ displays a more complex Raman spectrum than conventional TMDCs. In Figure 1c the Raman spectrum displays at least eleven Raman modes in the 100 ~ 300 cm^−1^ range, which is significantly more compared to other TMDCs with higher crystal symmetries. And exfoliated few-layer nanosheet has shown characteristic A_1g_ (out-of-plane) and E_2g_^1^ (in-plane) Raman modes located at 243 and 284 cm^−1^ for MoSe_2_, which proves the existence of Mo element. The few-layer Mo:ReSe_2_ nanosheets prepared by mechanical exfoliation are characterized by AFM. The thickness of these Mo:ReSe_2_ flakes is mainly in the range of 4–5 nm, which corresponds to a layer number of 7–8 (shown in Figure 1d). The energy-dispersive x-ray (EDX) is used to analyze the composition of the few-layer nanosheets, as shown in the Supporting Information (Figure S1).

**Figure 2:**
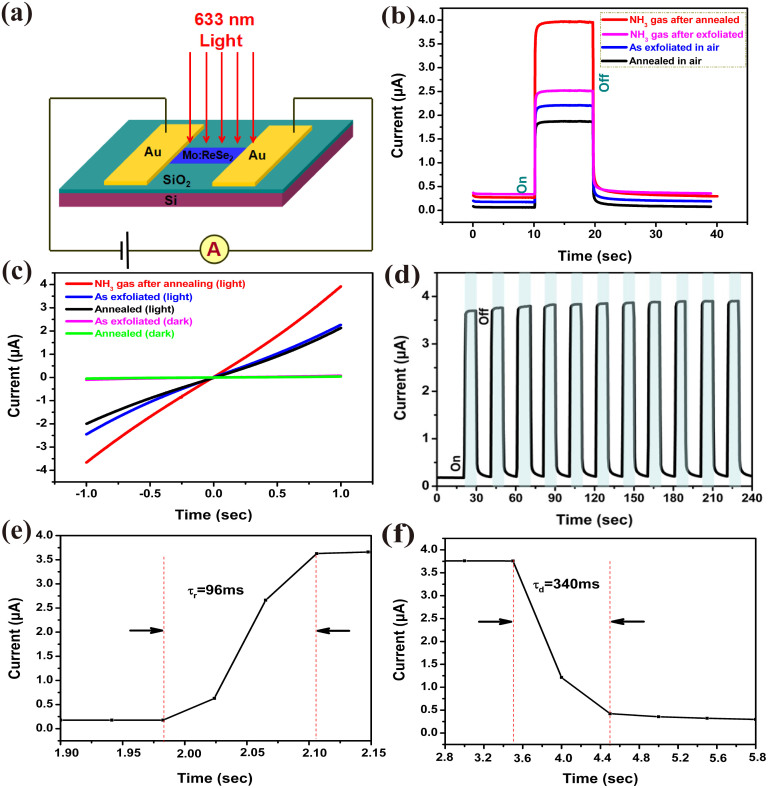
(a) Schematic of the device operation, (b) I-t curves and (c) I-V curves when the photodetector is illuminated with 633 nm light at an irradiance of 20 mW/cm^2^ under different conditions (the bias voltage between two electrodes is kept constant at 1 V), (d) multiple cycle operation of the device, (e) and (f) the photocurrent responses with time in the annealed photodectors under illumination of 633 nm in NH_3_ environment.

Two Au electrodes were made onto the few-layer Mo:ReSe_2_ nanosheet with a 28 μm wide channel. Monochromatic light (~633 nm) was vertically irradiated onto the device (depicted in Figure 2a). Figure 2b shows the photocurrent measured as a function of time (I-t curves) when the photodetectors are illuminated with irradiance of 20 mW/cm^2^, while the bias voltage between two electrodes is kept constant at 1 V. Under the light irradiation, the Mo:ReSe_2_ nanosheet strongly absorbs the photons which generate electron-hole pairs. The electron-hole pairs are separated by the external electric field, leading to the generation of photocurrent. Immediately after the light is turned on, the photocurrent rapidly rises and settles down to a highly stable and saturated value. In Figure 2b, a jump of ~2.2 μA is observed for the as-exfoliated device when the light is turned on in air. When the device is measured in NH_3_ environment, the dark current (I_dark_) is slightly increased, then after irradiated by light, a jump of 2.6 μA is obtained. And the photoswitch ratio (I_light_/I_dark_) of as-exfoliated device in NH_3_ is a little higher than that in air (Where I_light_ is photocurrent). It is illustrated that the as-exfoliated Mo:ReSe_2_ shows less sensitive to NH_3_ environment. To enhance the photosensitivity, an annealing process is needed for the as-exfoliated Mo:ReSe_2_ nanosheet. Here, the annealing can be regarded as a process to better expose the few-layer surface to the ambient by thermally driving away contaminants/organic residue, or it is possible that the annealing process might be creating a small density of chalcogen vacancies in the few layers (in Supporting Information Figure S2)^28^,^29^. After the annealing process, the photocurrent intensity of the nanosheet becomes extremely sensitive to gas environment. As shown in Figure 2b, the photocurrent of the annealed device measured in air decreases by ~0.5 μA. During exposed to NH_3_ environment, the photocurrent of the annealed device increases to ~4 μA, and the photoswitch ratio is about 20. Compared to the as-exfoliated ReSe_2_, the photoswitch ratio of the annealed Mo:ReSe_2_ is enhanced in NH_3_ environment. Current-voltage (I–V) characteristics measured in dark or under light illumination are shown in Figure 2c. All I–V curves both in dark and under light illumination are nearly linear, which indicates an Ohmic contact. In the dark, the I–V curve of as-exfoliated device is slightly different from annealed device. Under the light illumination, the photocurrent rises almost instantaneously. In addition, the annealed device exhibits a higher photocurrent when exposed in NH_3_ environment. Figure 2d shows the photocurrent switching of the device in NH_3_. Each photoresponse cycle consists of three transient regimes: sharp rise, steady state, and sharp decay. As shown in Figure 2d, with the light irradiation on and off, the current of the device exhibits a low-current state of 0.2 μA in the dark and a high-current state of ~4 μA under light illumination. After many cycles, the photocurrent still responds in a similar fashion to the light, which exhibits excellent operation reversibility and stability.

The spectrum responsivity (*R_λ_*) and EQE are two critical parameters to determine the sensitivity for an optoelectronic device, which represent the ability to provide photo-generated carriers per single incident photon. *R_λ_* and EQE can be expressed as *R_λ_ = ΔI/PS* and EQE = *t_life_/t_tran_* = *hcR_λ_/(eλ)*^19^,^30^, where *t_life_* and *t_tran_* are the lifetime of carriers and the charge transport time between electrodes, respectively; *ΔI* is the difference between the current under photo-excitation and the dark current; *P* is the light power intensity irradiated on the device (P = 20 mW/cm^2^); and *S* is the effective irradiated area of the device (S = 336 μm^2^). From our experimental results, under an illumination of 633 nm at 1 V (calculated from Figure 2d), the *R_λ_* and EQE are calculated to be ~55.5 AW^−1^ and ~10893%, respectively. Therefore, these photodetectors show a much better photoresponse compared to most other optoelectronic devices, as shown in Supporting Information Table 1.

The time response speed is also a key factor for photodetectors and it determines the capability of a photodetector to follow a fast-varying optical signal. Response times for photocurrent rise and decay obtained from 1D nanostructures based photodetectors range from seconds to several tens of minutes^30^. Figure 2e and 2f show the photocurrent responses in the annealed device under the illumination (633 nm) in NH_3_ environment. The dynamic response to the light illumination for rise and decay in our devices can be expressed by *I(t) = I_0_ [1 − exp(−t/τ_r_)]* and *I(t) = I_0_ exp(−t/τ_d_)*, τ_r_ and τ_d_ are the time constants for the rise and decay, respectively^31^,^32^. The rise time (decay time) is defined as the time interval for the response to rise (decay) from 10 to 90% (90 to 10%) of its peak value. The time constant τ_r_ and τ_d_ are calculated to be 96 ms and 340 ms, respectively, from our device.

## Discussion

**Figure 3:**
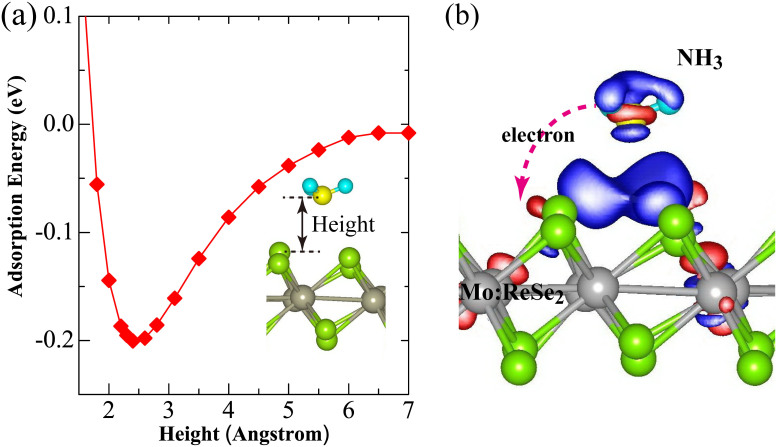
A NH_3_ molecule adsorbed on the Mo:ReSe_2_ monolayer. (a) Adsorption energy as a function of height between the N atom of NH_3_ and the topmost Re atom of Mo:ReSe_2_. The Inset shows the stable adsorption configuration. (b) Charge density difference. Red and blue correspond to charge accumulation and depletion, respectively. The isosurface value is set to be 6×10^4^ e/ Å^3^. The arrow indicates the direction of charge transfer between the NH_3_ and Mo:ReSe_2_.

In order to understand the experimental results, first-principles calculations are performed to investigate the adsorption of NH_3_ molecule on the Mo:ReSe_2_ surface. A 4 × 4 supercell of Mo:ReSe_2_ monolayer with a single NH_3_ molecule adsorbed to it is built for the calculation. The calculated adsorption energy curve for NH_3_ in Figure 3a shows that the interaction between the NH_3_ molecule and Mo:ReSe_2_ monolayer can be characterized as physisorption due to the small adsorption energy and large separation distance. At equilibrium state, the adsorption energy and separation distance are found to be −203 meV and 2.34 Å, respectively. The charge transfer between NH_3_ molecule and Mo:ReSe_2_ monolayer is then determined by using the Bader analysis method. It is found that NH_3_ molecule behaves as a charge donor and donates approximately 0.024 electrons (per supercell) to the underlying Mo:ReSe_2_ monolayer, depleting the charge on NH_3_ molecule, as shown in Figure 3b. Since the mechanically exfoliated Mo:ReSe_2_ nanosheet is a n-type semiconductor with background free electrons coming probably from defects (as shown in Supporting Information Figure S3), the adsorption of NH_3_ further transfers electrons to the nanosheet and increases its carrier density^29^,^33^,^34^. Point defects in 2D materials can trap free charge carriers and localize excitons^28^. The charge transfer value can be enlarged if the adsorption of NH_3_ molecule occurs at a defect site induced by the experimental annealing. For instance, when NH_3_ is adsorption at a single Se vacancy, a larger value of 0.049 electrons can be transferred from the NH_3_ molecule to the monolayer Mo:ReSe_2_ (as shown in Supporting Information Figure S4). So we propose the mechanism in the following. After exposed to light, photo-generated carriers produce in Mo:ReSe_2_ nanosheet and move in direction under external electric field, resulting in the generation of photocurrent. When the photoresponse is measured in NH_3_ environment, more carriers can be produced than that in air. So we can find that the photoresponse of as-exfoliated device in NH_3_ is slightly better than that in air. To activate the NH_3_ molecule adsorption, the annealing is a necessary process because of more point defects can be created, resulting in more charges transfer. Therefore, the annealed device exhibits much better photosensitive property in NH_3_ environment.

In summary, photodetectors fabricated from few-layer Mo:ReSe_2_ nanosheets have been demonstrated. The photoresponse and EQE of the photodetectors were measured at different gas environments and shown to reach ~55.5 AW^−1^ and 10893%, respectively, under 633 nm light irradiation in NH_3_ environment. The switching of photocurrent was quick and stable. A theoretical investigation of the effect of NH_3_ on the enhanced photoresponse was also performed. These experimental and theoretical findings indicate that as a new 2D TMDC, Mo:ReSe_2_ nanosheet could be an excellent candidate for high-performance nanoscale sensors, photoelectronic switches and optoelectronic circuits.

## Methods

### Crystal growth

Single crystals of the Mo:ReSe_2_ were grown using the chemical vapour transport method with Br_2_ as a transport agent containing two step growth process, leading to n-type conductivity^33^. Prior to the crystal growth, a quartz tube (20 cm length) containing Br_2_ (~5 mg cm^−3^), Mo (99.99%), Re (99.99%) and Se (99.999%) was cooled with liquid nitrogen, then evacuated and sealed. After that, the powder was shaken well to achieve uniform mixing. The quartz tube was placed in a two-zone furnace and it pre-reacted at 850°C for 24 h with the growth zone temperature at 1000°C to prevent the transport of the product. The furnace was then adjusted to one zone at 1000°C with another zone at 1060°C, and was produced the temperature gradient over 24 h. With the temperature varying from 1060 to 1000°C, a temperature gradient of approximately 2°C cm^−1^ over an ampoule length of 20 cm gave optimal conditions for the single-crystal growth of the Mo:ReSe_2_. After 360 h crystallization, the furnace was allowed to cool down slowly (40°C h^−1^) to about 200°C. Then the ampoule was taken out and the temperature of the end away from the crystals was dropped to condense the Br_2_ vapor. When the ampoule reached room temperature, it was opened and the crystals removed. The crystals were then rinsed with acetone and deionized water.

### Mechanical Exfoliation of Mo:ReSe_2_

Few-layer Mo:ReSe_2_ nanosheets were isolated from bulk Mo:ReSe_2_ single crystals and then deposited onto the freshly cleaned Si substrates covered by a 300 nm thick SiO_2_ layer using the scotch tape-based mechanical exfoliation method, which was widely employed for preparation of single-layer graphene sheets^21^.

### Thermal Annealing

The samples were heated to 400°C in a 25°C/min rate and the temperature was held at 400°C for one hour in vacuum. After annealing, the furnace was cooled down to room temperature and the samples were taken out of the furnace.

### Photodetector devices were fabricated as follows

The Au electrodes were thermally evaporated by laying a Au wire with micrometer-sized diameter as the mask on Mo:ReSe_2_ nanosheet exfoliated on the Si/SiO_2_ substrates to obtain a gap between two electrodes. After the deposition of electrodes, the Au wire mask was removed so that the sample in gap area could be exposed. Electrochemical experiments were all performed with a CHI660D electrochemical workstation in a conventional three-electrode electrochemical cell.

### Raman Spectroscopy

Analysis of the few-layer Mo:ReSe_2_ nanosheet by Raman spectroscopy was carried out on a WITec CRM200 confocal Raman microscopy system with the excitation line of 532 nm and an air-cooling charge-coupled device (CCD) as the detector (WITec Instruments Corp, Germany).

### EDX analysis

EDX was utilized for the estimation the composition of Mo:ReSe_2_. The concentration of Mo in the ReSe_2_ crystals was shown in supporting information Figure S1.

### Computational Methods

First-principles calculations are performed using the Vienna *ab initio* simulation package (VASP)^35^,^36^ on the basis of density-functional theory (DFT). The exchange-correction interaction is treated by the van der Waals density functional (vdW-DF)^37^,^38^ to describe this adsorption system. Meanwhile, a cutoff energy of 450 eV and a Monkhorst-Pack grid^39^ of 5 × 5 × 1 for the Brillouin zone integration are employed. In order to eliminate the interaction between two adjacent Mo:ReSe_2_ monolayers, a vacuum layer larger than 15 Å is adopted. The geometric structure is fully relaxed until the Hellmann-Feynman force on each atom is less than 0.02 eV/Å. By means of Bader analysis^40^, charge transfer between Mo:ReSe_2_ and NH_3_ molecule is obtained. The adsorption energy is defined as 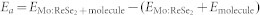, where 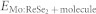, 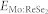 and *E*_molecule_ are the total energies of molecule adsorbed Mo:ReSe_2_, bare Mo:ReSe_2_ and isolated molecule. Negative value of E_a_ indicates an exothermic adsorption process. The charge density difference is calculated by the formula, 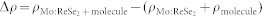, where 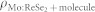, 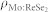 and *ρ*_molecule_ are the charge densities of molecule adsorbed Mo:ReSe_2_, bare Mo:ReSe_2_ and isolated molecule, respectively.

## Author Contributions

S.Y. conceived the project. S.Y., S.T., Y.L., B.L. and F.L. performed the synthesis and measurements. Q.Y. performed the density functional theory calculations. S.Y. wrote the manuscript. All authors have read the manuscript.

## Supplementary Material

Supplementary Information Supporting information
