# Supplementary material for: High-Performance Few-layer Mo-doped ReSe2 Nanosheet Photodetectors
Source: Sci Rep. 2014 Jun 25;4:5442. doi: 10.1038/srep05442 (PMC4069702; doi:10.1038/srep05442)
Supplement: Supplementary Information — Supporting information [file srep05442-s1.doc]

High-Performance Few-layer Mo-doped ReSe_2_ Nanosheet Photodetectors

Shengxue Yang^1,*^, Sefaattin Tongay^1,2^, Qu Yue^3^, Yongtao Li^1^, Bo Li^1^, Fangyuan Lu^1^

^1^State Key Laboratory of Superlattices and Microstructures, Institute of Semiconductors, Chinese Academy of Sciences, P.O. Box 912, Beijing 100083, China

^2^School for Engineering of Matter, Transport and Energy, Arizona State University, Tempe, AZ 85287, United States

^3^College of Science, National University of Defense Technology, Changsha 410073, China


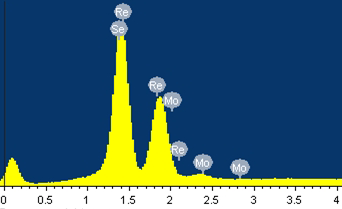

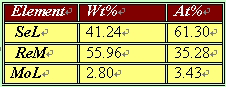


Figure S1 The EDX of the sample.


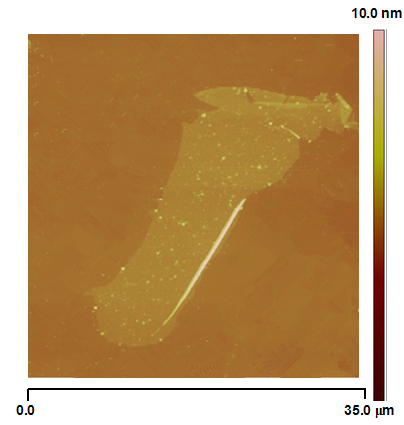


Figure S2 AFM of the annealed Mo:ReSe_2_ nanosheet.


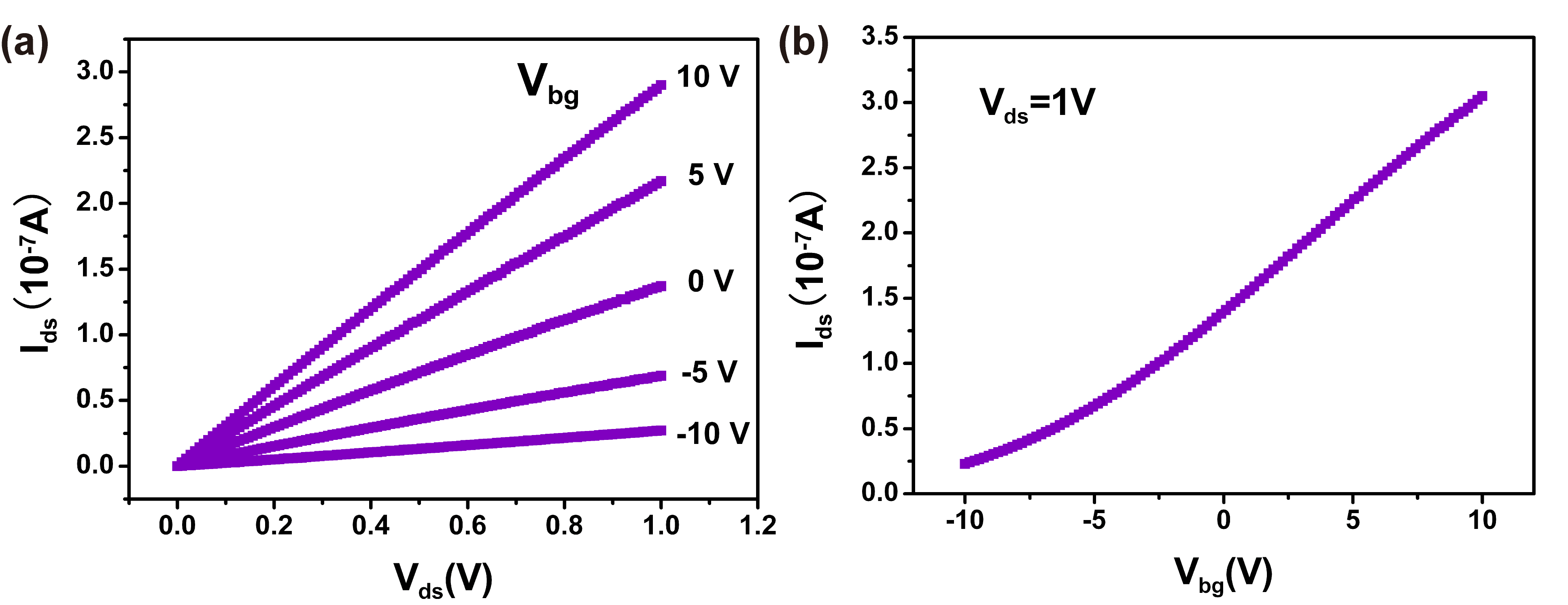


Figure S3 (a) Output characteristics of few-layer Mo:ReSe_2_ transistors in dark recorded for different back-gate voltages V_bg_ (-10~10V), (b) Room-temperature transfer characteristic curve of few-layer transistors with 1V applied bias voltage V_ds_.


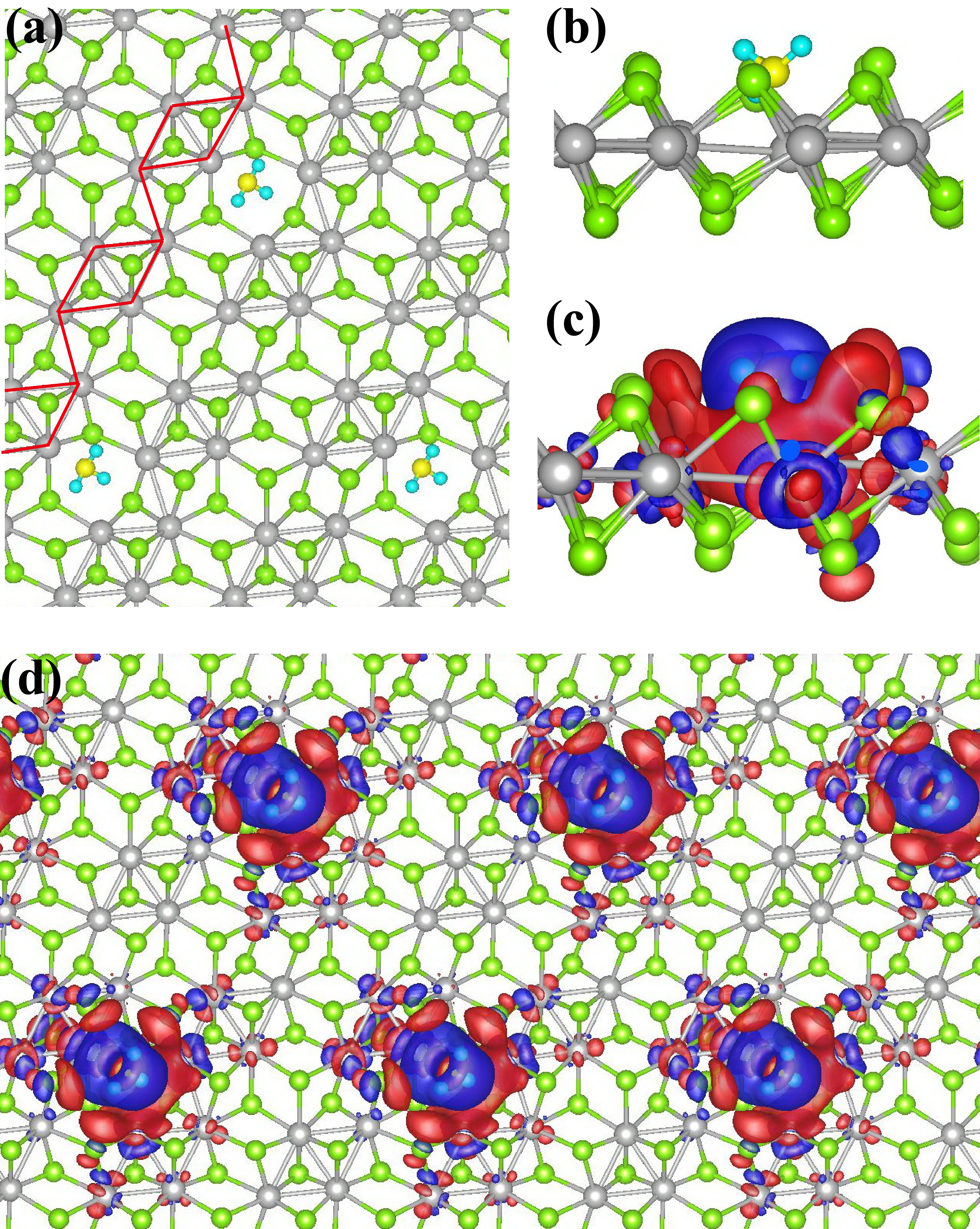


Figure S4 Adsorption of NH_3_ molecule on monolayer Mo:ReSe_2_ at the Se vacancy site. (a) The top view and (b) side view of adsorption configuration, (c) the side view and (d) top view of charge density difference. Red and blue distributions correspond to charge accumulation and depletion, respectively. The isosurface value is set to be 6×10^4^ e/ Å^3^.

Table 1 Comparison of the parameters of our device to the reported 2D material based photodetectors.

| **Photodetectors** | **R*λ* (AW^-1^)** | **EQE (%)** | **Response**  **time** |
| --- | --- | --- | --- |
| Single-Layer MoS_2_ ^[1]^ | 7.5×10^-3^ |  | 50 ms |
| Graphene ^[2]^ | 1.0×10^-3^ | 6-16 |  |
| Multilayer MoS_2_ ^[3]^ | 9.0×10^-5^ |  |  |
| Graphene ^[4]^ | 6.1×10^-3^ | 1500 |  |
| GaS Nanosheet ^[5]^ | 4.2 | 2050 | ~30 ms |
| Our photodetector | 55.5 | 10893 | ~96 ms |

**References**

1. Z. Yin, H. Li, H. Li, L. Jiang, Y. Shi, Y. Sun, G. Lu, Q. Zhang, X. Chen, H. Zhang, *ACS Nano* **6**, 74 (2012).

2. F. Xia, T. Mueller, Y. Lin, A. Valdes-Garcia, P. Avouris, *Nat. Nanotechnol.* **4**, 839 (2009).

3. W. Choi, M. Y. Cho, A. Konar, J. H. Lee, G. Cha, S. C. Hong, S. Kim, J. Kim, D. Jena, J. Joo, S. Kim, *Adv. Mater.* **24**, 5832 (2012).

4. Y. Liu, R. Cheng, L. Liao, H. Zhou, J. Bai, G. Liu, L. Liu, Y. Huang, X. Duan, *Nat. Commun.* **2**, 579 (2011).

5. P. Hu, Z. Wen, L. Wang, P. Tan, K. Xiao, *ACS Nano* **6**, 5988 (2012).
